# Cancer-associated fibroblasts: tumor defenders in radiation therapy

**DOI:** 10.1038/s41419-023-06060-z

**Published:** 2023-08-22

**Authors:** Yalin Zhang, Na Lv, Manshi Li, Ming Liu, Chunli Wu

**Affiliations:** 1 Department of Radiation Oncology, Fourth Affiliated Hospital of China Medical University, Liaoning, China; 2 Department of Clinical Epidemiology, Fourth Affiliated Hospital of China Medical University, Liaoning, China

**Keywords:** Cancer microenvironment, Cancer therapy

## Abstract

Cancer-associated fibroblasts (CAFs) are an important component of the tumor microenvironment that are involved in multiple aspects of cancer progression and considered contributors to tumor immune escape. CAFs exhibit a unique radiation resistance phenotype, and can survive clinical radiation doses; however, ionizing radiation can induce changes in their secretions and influence tumor progression by acting on tumor and immune cells. In this review, we describe current knowledge of the effects of radiation therapies on CAFs, as well as summarizing understanding of crosstalk among CAFs, tumor cells, and immune cells. We highlight the important role of CAFs in radiotherapy resistance, and discuss current and future radiotherapy strategies for targeting CAFs.

## Facts

- Cancer-associated fibroblasts (CAFs) affect multiple stages of cancer progression.
- Radiation therapy caused the phenotypic, secreted, and gene expression alterations in CAFs.
- CAFs play adverse effects in cancer radiation therapy (RT).

## Open questions

- Different RT doses and fractionation regimens may have different effects on CAF function. How should we choose the right radiotherapy regimen?
- CAFs are a functionally heterogeneous population in the TME, and the activation conditions vary across the heterogeneous populations. How can we use it to develop more effective treatment strategies?
- What is the future direction of cancer therapy based on CAFs?

## Introduction

Stephan Paget first proposed the “seed and soil” theory, which proposes that tumor cells are “seeds” that depend on the surrounding microenvironment, “soil”, for their development and metastasis [1]. Today, in the field of oncology, cancer cell-centered perspectives have evolved into a more wholistic view of cancer, including cancer cells, other non-malignant cells, and non-cellular components, known as the tumor microenvironment (TME). It is inadequate for research efforts to improve cancer treatment to focus solely on cancer cells carrying genetic mutations; the TME must also be considered as its interactions with tumor cells frequently contribute to malignant disease occurrence, development, and response to treatment. The TME is a dynamic network composed of tumor cells, infiltrating immune cells, and CAFs, as well as factors secreted by these cells and non-cellular components of the extracellular matrix (ECM) [2]. Heterogeneous cell populations in the TME, with different or overlapping functions, are involved in tumorigenesis, among which fibroblasts play a crucial role [3]. CAFs, a vital cellular component of the TME, provide functional assistance and structural support for tumor progression and metastasis through their interactions with tumor cells [4]. CAFs have been detected in the stroma of various cancers and their presence has been associated with poor prognosis of patients with several types of solid tumors [5, 6].

RT is a cornerstone of oncology treatment, with more than half of all cancer patients undergoing RT to cure localized tumors [7], relieve symptoms, or control tumor progression. The primary antitumor effect of RT is induction of tumor cell death, but recent findings suggest that RT also rapidly and consistently alters the TME [8], and enhances systemic responses to cancer immunotherapy [9–11]. RT has demonstrated effective tumor control in several preclinical and clinical models and is proven to exert immunostimulatory effects by inducing immunogenic cell death, releasing tumor-associated antigens, and triggering an abscopal effect [12–14]. Early clinical data support that combination RT and immunotherapy modulates the TME and can be dose-dependent, providing superior better local and systemic tumor control [10, 15, 16].

The impacts of RT on CAFs vary among studies, and the possible effects and mechanisms underlying the influence of CAFs on tumor radioresistance remain controversial. Nevertheless, appealing targets for therapeutic intervention can only be designed if more precise and detailed understanding of the role of CAFs in RT is achieved. In this review we present an overview of the results on studies of the effects of RT on CAFs, as well as its indirect influence on the TME through CAFs.

## Definition and origin of CAFs

In the context of cancer, the definition of CAFs can be simply stated as the mesenchymal fibroblastic component of the TME [17]. As the most abundant component of the TME, CAFs are generally considered to be all fibroblasts found within and around tumor tissue. When analyzing tissue biopsy samples, the simplest view is that primary CAFs should be negative for epithelial (EpCAM), endothelial (CD31), and leukocyte (CD45) markers, have an elongated spindle-like morphology and lack mutations found within cancer cells [18]. Most studies have identified CAFs by asswssing for the expression of “CAF markers” including fibroblast activation protein and alpha-smooth muscle actin, which are thought to distinguish CAFs from normal fibroblasts [17]; however, these markers are not exclusive to CAFs, and no pan-specific CAF markers or uniform definitions have been identified thus far. A combination of morphology and markers may be the most reliable method to identify CAFs.

A significant source of CAFs is normal tissue-resident fibroblasts, also referred to as quiescent fibroblasts, activated by surrounding tumor blasts [19]. Distinct from normal fibroblasts, activated CAFs isolated from various human tumors exhibit increased autocrine sign transduction capacity, proliferative tendencies, and are metabolically active [20]. Within the TME, activated CAFs also secrete cytokines, growth factors, as well as producing ECM proteins and enzymes involved in ECM remodeling [21]. Numerous studies have reported that CAFs may be also derived from smooth muscle cells, endothelial cells, adipocytes, pericytes, bone marrow-derived stem cells, and even epithelial cells [22–26]. Given the diversity of their cellular predecessors, CAF populations are complex, with various fibroblast phenotypes, and diverse functions in many cancer types [27]. A significant challenge is to understand the extent to which this heterogeneity serves in their diverse biological responses to cancer cells. Development of methods to distinguish CAF subpopulations, based on expression levels of numerous biomarkers, as well as spatial and temporal single-cell transcriptome and proteome analyses, may facilitate deeper characterization of CAFs.

## Impact of radiation on CAFs

### CAFs exhibit unique radioresistance

Upon exposure to ionizing radiation, CAFs exhibit unique radioresistance, as radiation has cytotoxic, but not cytolytic, consequences for these cells [28]. Small doses of radiation may not critically alter the phenotype and features of CAFs, whereas CAFs become senescent within days after large radiation doses, resulting in decreased motility and reduced proliferation [29]. The decreased motility of CAFs after irradiation exposure may attributable to increased expression of integrins, particularly β1-integrin. In a study by Adamantia Papadopoulou et al. a single radiation dose (4 Gy) quickly induced a DNA damage response, prompting serious, but reversible, cell cycle arrest. After a sequence of doses (total ~50 Gy) cellular senescence accelerated,enduring growth arrest occured, and specific biochemical and morphological senescence-related markers were upregulated; this process was considered p53-dependent [30]. In rectal cancer, CAFs are polarized toward an inflammatory phenotype and trigger oxidative DNA damage on irradiation, thus predisposing inflammatory CAFs to p53-mediated treatment-induced senescence, which in turn leads to chemoradiation resistance and disease progression; this process is related to IL-1α [31]. These findings suggest that RT influences the CAF phenotype, which may support tumor cell growths.

Although there is already considerable evidence supporting the radioresistance of CAFs, the underlying mechanism remains unknown. New evidence suggests that radiation resistance largely depends on the tissue of origin of CAFs [32]. Researcher found that CAFs of skin (AG1522) and lung (MRC5) were more resistant to ionizing radiation than ordinary fibroblasts. Specifically, AG1522 CAFs were more resistant to 137Cs γ rays after co-culture with different types of cancer cell. Fuether, CAFs developed radioresistance after co-culture with cancer cells, but not with non-malignant epithelial cells. Notably, CAF radioresistance is associated with a greater potential to restore single- and double-strand DNA breaks, as well as increased oxidative stress levels. Addition of single- or double-stranded DNA repair inhibitors attenuated CAF resistance to the clastogenic consequences of γ rays. In conclusion, the metabolism, epigenetic modifications, and tissue origin of CAFs in the TME can all influence their response to ionizing radiation.

### Radiation affects CAF secretory function

As important components of the TME, CAFs secrete various soluble cytokines, chemokines, and other factors, including CXCL12, vascular endothelial growth factor (VEGF), and transforming growth factor β (TGF-β) through various paracrine mechanisms, which have diverse effects on tumor phenotype, and can have pro-tumor properties [33]. The secretory functions of CAFs are affected by RT; expression of many growth factors and chemokines is increased in response to RT, where the most variable is TGF-β. The clear increase in TGF-β expression is believed to be due to increased fibroblast activation [34]. In addition to its established immunosuppressive function against inflammation and immune cells [35], TGF- β regulates ECM synthesis and tissue rigidity, thereby causing both direct and indirect immunomodulatory effects [36, 37]. Furthermore, increased expression of cell surface proteoglycan synthesis sugar 1 is also suggested to be associated with TGF-β autocrine activity through the SMAD pathway and the transcription factor SP1, which in turn can promote tumor growth and angiogenesis [38]. Similarly, levels of secretion of cytokines, such as CXCL1, CXCL-12, IGF1, IGF2, and β-hydroxybutyrate, by CAFs were increased in response to ionizing radiation. The crosstalk between CAFs and other cells in the TME mainly occurs through cytokine and chemokine secretion, and the influence of secretory changes on tumor progression after RT will be detailed in the following sections.

### Altered gene expression of CAFs after radiation

To better understand the collateral outcomes of ionizing radiation on normal tissues and tumor cells, several researchers have attempted to decode global changes in gene expression in normal fibroblasts and CAFs using gene microarray technologies [39–42]. CAF transcriptome analysis 24 h after radiation treatment revealed a total of 680 differentially expressed genes in irradiated CAF samples, of which 127 were downregulated and 553 were upregulated [39]. Analysis of these data showed that various genes involved in cell cycle regulation and DNA damage repair, such as cell division control protein 6 homolog and the DNA mismatch repair protein, MSH2, were upregulated. Genes involved in pro-apoptotic and anti-apoptotic signaling were also altered, and the cellular consequences were validated by PCR and in vitro experiments. Furthermore, differentially expressed genes were associated with mechanisms involved in cellular stress responses and other pro-survival pathways. We infer that ionizing radiation induces severe genotoxic stress in cells, accompanied by changes in gene expression associated with biological activities including DNA repair, cell proliferation, cell cycle, apoptosis, and the p53 pathway [39], consistent with a previous study of human primary skin fibroblasts [43].

## CAFs regulate tumor cell biological functions

**Fig. 1:**
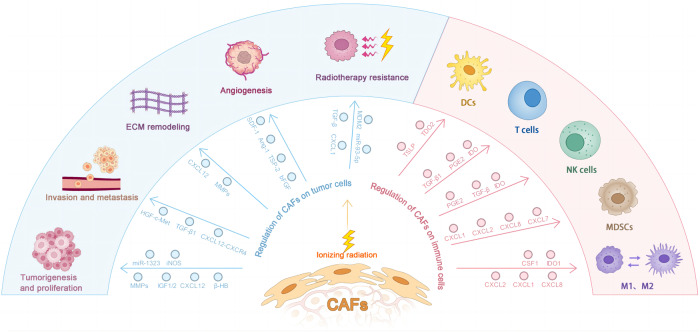
Cancer-associated fibroblasts modulate the tumor microenvironment through the secretion of many cytokines and chemokines. After radiotherapy, CAFs are involved in multiple links of tumor development, including tumorigenesis and proliferation, invasion and metastasis, ECM remodeling, angiogenesis, and resistance to radiotherapy. In addition, CAF maintains its immunosuppressive capacity after receiving ionizing radiation. Abbreviation: PGE2 prostaglandin E2, TDO2 tryptophan csf-dioxygenase, TSLP thymic stromal lymphopoietin.

The interplay among tumor and stromal cells in the TME is considered a significant driver of tumor development and metastasis [44]. As the most abundant stromal cells in the TME, CAFs have key roles in cancer progression [20]. Through cell-cell contacts, release numerous regulators, and remodeling the ECM, CAFs regulate the biology of tumor cells and other stromal cells, and these cells subsequently influence cancer initiation and development [45]. The interplay between tumor cells and CAFs during RT is vital to the biological traits of tumor cells and for clinical tumor RT. Here, we provide representative but not exhaustive. examples of aspects of the association of irradiated CAFs with tumor cell functions. (Table 1) (Fig. 1).

**Table 1:** Summary of studies on irradiated CAFs regulating the biological functions of tumor cells.

Effect of CAFs	Tumor type	Results	Possible mechanisms	Reference
CAFs in tumorigenesis and proliferation	Lung	Increased survival	Increased expression of MMPs	[30]
	Lung and melanoma	Increased recurrence and survival	IGF1/2, CXCL12 and β-HB produced by CAFs induced autophagy	[47]
	Cervical cancer	Increased proliferation, migration, invasion, and radiosensitivity	Exosomes secreted by CAFs increased miR-1323 secretion	[48]
	PDAC	Promoted growth	A substantial increase in iNOS secreted by CAFs	[49]
CAFs in invasion and metastasis	PDAC	Promoted invasion	The levels of HGF remained unchanged, but the phosphorylation of the HGF receptor was increased	[52]
		Promoted invasion	Increased the expression of CAFs to CXCL12	[85]
	ESCC	Promoted invasion	HGF and β-catenin expression were increased, which can increase the phosphorylation of c-Met and MAPK activity	[51]
	SCC	Promoted invasion and growth	Enhancement of invasive growth-related molecules such as c-Met, Ras and laminin 5 and filamin A through TGF- β1-mediated bystander mechanism	[53]
ECM remodeling		Increased the amount of ECM protein	Increasd the number and activity of fibroblasts	[55]
		Catabolizes ECM	Increased the expression of MMPs	[55]
CAFs in angiogenesis	NSCLC	Reduced their pro-angiogenic properties	SDF-1, Ang-1, and TSP-2 were all downregulated, whereas the release of bFGF was upregulated	[58, 59]
CAFs promote radiotherapy resistance	PDAC	Enhanced radioresistance	Enhanced the CSC phenotype, as TGF-β participating	[63]
	CRC	Enhanced CSC clonogenicity and radioresistance	Exosomes derived from CAFs activated TGF-β signaling pathway	[64]
	Rectal cancer	Enhanced radioresistance	CAFs-derived exosomes contained higher miR-93-5p	[65]
	NSCLC	Enhanced radioresistance	The nine different MDM2 transcripts showed elevated expression levels	[39]
	ESCC	Enhanced radioresistance	Increased the expression of CAFs to CXCL1	[66]
	Nasopharyngeal carcinoma cells	Enhanced radioresistance	Increased the expression of CAFs to IL-8	[67]

Abbreviations: *Ang-1* angiopoietin-1, *bFGF* basic fibroblast growth factor, *CAFs* cancer-associated fibroblasts, *CRC* colorectal cancer, *CSC* cancer stem cell, *ECM* extracellular matrix, *ESCC* esophageal squamous cell carcinoma, *HGF* hepatocyte growth factor, *iNOS* inducible nitric oxide synthase, *MMP* matrix metalloprotease, *NSCLC* non-small cell lung carcinoma, *PDAC* pancreatic ductal adenocarcinoma, *SCC* squamous cell carcinoma, *SDC1* cell surface proteoglycan synthesis sugar 1, *SDF-1* stromal cell-derived factor 1, *T* thrombospondin-2, *β-HB* β-hydroxybutyrate.

### CAFs in tumorigenesis and tumor cell proliferation

RT can affect the tumor-promoting function of CAFs in the TME. CAFs influence tumor cell survival both before and after RT, although the underlying mechanisms differ. The effects of CAFs on tumor growth after RT are primarily achieved by altered secretion. Adamantia et al. [30] investigated the impact of growth of human lung cancer cell lines in the presence of prematurely senescent CAFs and found that irradiation-mediated senescent CAFs significantly enhanced cancer cell proliferation in immunodeficient mice. A specific matrix metalloprotease (MMP) inhibitor significantly reduced cancer growth in the presence of senescent fibroblasts; hence, this effect may be associated with increased expression of MMPs; however, in another study, administration of high-dose γ radiation (44 Gy) did not increase in MMP expression in breast cancer fibroblasts. Theese different findings may be related to variations in the radiation regimens and tissue types investigated [46]. Wang et al. [47] demonstrated that IGF1 / 2, CXCL12, and β -hydroxybutyrate produced by CAFs suppress mTOR activity, which subsequently induces cancer cell autophagy following radiation treatment, and promotes the recovery of cancer cells with radiation-induced damage in vitro and in vivo in mice. They also retrospectively analyzed recurrence and survival times in patients with lung cancer and hepatocellular carcinoma receiving conventional external beam RT (EBRT) and stereotactic body RT (SBRT). SBRT is a focused form of radiation therapy that delivers high doses of radiation directly to the tumor in a few sessions. It uses advanced imaging technology to precisely target the tumor and spare healthy surrounding tissues. Compared to SBRT, conventional EBRT delivers a lower dose of radiation per session but can cover a larger area. That is, SBRT can map more precisely to the tumor tissue while having less effect on the surrounding CAFs. It was greatly inferior to EBRT in terms of patient survival and recurrence, due to its lack of effects on peripheral CAFs, further supporting speculation that CAFs promote cancer cell recovery and tumor recurrence after RT. In a study on cervical cancer [48], miR-1323 secretion from CAFs in exosomes was increased, while miR-1323 downregulation suppressed cell proliferation, migration, invasion, and increased cell radiosensitivity in cervical cancer. Furthermore, CAFs can promote the growth of pancreatic ductal adenocarcinoma (PDAC) cell growth, and this effect increases when cells are cultured in conditioned media from RT-treated CAFs due to a substantial increase in inducible nitric oxide synthase (iNOS) secretion by CAFs after RT, which can be eliminated in vitro by iNOS depletion using genetic and pharmacological approaches [49].

### CAFs in cancer invasion and metastasis

The effects of CAFs on invasion and metastasis of most cancers primarily occur through ECM remodeling, regulation of epithelial-to-mesenchymal transition (EMT), secretion of cancer growth factors, and effect on treatment response [50]. This is principally accomplished by signaling among CAFs, cancer cells, and ECM exerted via cell-cell contacts, cytokine secretion, or exosomes. Here, we describe the effects of CAFs tumor invasion and metastasis through cytokine secretion and EMT regulation following RT. Co-culture of nonirradiated fibroblasts significantly increased the invasion capacity of PDAC and esophageal squamous cell carcinoma (ESCC) cells [51, 52], as demonstrated by in vitro invasion assays. Surprisingly, the increased invasiveness was further accelerated when cells were co-cultured with irradiated fibroblasts. Levels of hepatocyte growth factor (HGF) remained unchanged in pancreatic cancer cells grown in irradiated CAF-conditioned medium, but phosphorylation of the HGF receptor was increased in tumor cells cocultured with CAFs [52]. In contrast, HGF and β-catenin expression were increased in ESCC cells in the irradiated group in both in vitro and vivo models [51]. HGF secreted in response to CAF irradiation can enhance the phosphorylation activity of c-MET and MAPK in tumor cells, which translates into stronger invasion capacity. Hence, overactivity HGF-c-MET axis, triggered by CAF responses to RT, stimulates tumor progression. Alternatively, irradiated CAFs can promote invasion and growth of squamous cell carcinoma (SCC) cells by stimulating invasive growth-related molecules such as c-MET, RAS, Laminin 5 and Filamin A through a TGF- β1-mediated bystander mechanism, possibly involving irradiated CAF-induced genomic instability of SCC cells [53]. Another study on pancreatic cancer showed that radiation stimulates CAFs to secrete high concentrations of CXCL12 and act on pancreatic cancer cells through CXCR4, directly promoting EMT and tumor cell invasion. Further, the investigators found that CXCL12-CXCR4 signaling promotes EMT and invasion of pancreatic cancer cells by activating the P38 pathway.

### ECM remodeling

Tumor ECM, comprising basement membrane and intercellular stroma, is a dynamic structure that is remodeled during tumor progression and treatment. ECM remodeling in the TME occurs frequently in tumor tissues and, in most cancers, is generally associated with progression. CAFs are important stromal cells that are the major producers of ECM components such as collagen [54]. RT not only changes the phenotype of CAFs, but also increases their numbers and activity, thus increasing the amount of ECM proteins produced byfibroblasts, and physically preventing immune cells from accessing the TME, which is associated with poor recurrence-free survival. In addition, RT can increase the expression of MMPs, which catabolize the ECM, increasing cancer spread and metastasis [55].

### CAFs in angiogenesis

Aberrant angiogenesis is a key mediator of cancer development. As well as modulating the ECM, CAFs can directly promote tumor progression by secreting CXCL12 and VEGF-A [56, 57], which promote angiogenesis; however, the proangiogenic function of CAF is influenced by RT. Hellevik et al. [58] investigated the effect of CAFs on angiogenesis in non-small cell lung carcinoma (NSCLC) after ablative doses of radiation. Exposure to a single excessive dose of radiation (18 Gy) triggered changes in the profile of secreted signaling molecules involved in inflammation, angiogenesis, and tumor growth in lung CAFs. Expression levels of stromal cell-derived factor 1, angiopoietin-1, and thrombospondin-2 were increased, while those of basic fibroblast growth factor were decreased. In vitro, CAF-conditioned medium did not affect tumor cell proliferation and migration potential; however it decreased endothelial cell migration capacity to some extent, suggesting that exposure of CAFs to high radiation doses substantially reduces their proangiogenic properties; Grinde et al. [59] reported similar changes in CAF secretion. Furthermore, tumors established with fractionated-irradiated CAFs (3 × 6 Gy) had higher microvascular density than those established with CAFs exposed to a single high dose of radiation (1 × 18 Gy). These findings do not exclude the possibility that radiation may also trigger pro-angiogenic responses through tumor components other than CAFs.

### CAFs promote radioresistance

Despite several advances in RT over previous decades, radioresistance remains an unresolved issue in cancer therapy [60]. Given the substantial proportion of CAFs in tumor tissues, it is imperative to determine the role of fibroblasts in radioresistance. While the underlying mechanisms by which CAFs promote radioresistance are the subject of ongoing exploration, it is currently believed that various growth factors and pathways have significant roles in this process. CAF-conditioned medium enhances the cancer stem cell (CSC) phenotype and radioresistance of pancreatic cancer cells [61–63]. Proteomic screening revealed that TGF-β participates in the radioresistant phenotype, while addition of TGF-β-neutralizing antibodies repressed both EMT and CSC phenotypes, consequently sensitizing cancer cells to RT and decreasing tumorigenicity in vivo. Likewise, radioresistance observed in patients with colorectal cancer (CRC) may be related to the presence of CSCs. CRC cells were shown to exhibit stemness and radioresistance when cultured in CAF-conditioned medium, due to exosomes from CAFs activating TGF-β signaling [64]. Moreover, CAF-derived exosomes contained higher miR-935p levels than normal fibroblasts, which reduced radiation-induced apoptosis in SW480 cells [65]. The underlying mechanism may involve downregulation of FOXA1 and upregulation of TGFB3 on exosomes. In a study on ESCC, Zhang et al. [66] found that expression of the human chemokine, CXCL1, was much higher in CAFs than in normal fibroblasts. CXCL1 inhibits expression of the reactive oxygen species (ROS)-scavenging enzyme, superoxide dismutase 1, resulting in increased ROS accumulation after radiation, thus enhancing DNA damage repair and mediating radioresistance. CXCL1 also influences the effects of radioactivity through activation of the MEK/ERK pathway. Inhibition of CXCL1 expression by CAFs significantly reversed the radioresistance conferred by CAFs, both in vitro and in vivo. Similarly, CAFs promoted radioresistance and reduced the levels of DNA damage in nasopharyngeal carcinoma cells by secreting IL-8, which activates NF-κB signaling [67]. Altered transcriptional profiles of CAFs also contribute to radioresistance. Martinez-Zubiaurre et al. [39] conducted transcriptomic analysis of CAFs after RT in an NSCLC model study, and found that nine different *MDM2* transcripts all exhibited elevated expression levels. Previous studies have established that increased expression levels of MDM2 variants are associated with radiosensitivity in the lung [68], implying that CAF may partially mediate radioresistance after treatment.

## CAF regulation of immune cells

Recently, the interplay between CAFs and immune cells in the TME has attracteed more attention. In addition to their part in controlling disease cell activity, CAFs have key roles in molding the TME towards an immunosuppressive milieu, as well as contributing to tumor progression, by increasing immunosuppressive cytokine production and enhancing immune checkpoint expression [69]. The interaction of CAFs and the tumor immune microenvironment has vital roles in advancing tumor development. The association of CAFs with immune cells in cancer primarily leads to immune surveillance [70] and immune evasion of tumor cells [71]. An important question is, ‘Does RT affect the interaction between CAFs and tumor cells?’ Here we summarize current understanding of the impact of irradiated CAFs on immune cell populations in the TME. (Fig. 1).

### Dendritic cells

Dendritic cells (DCs) are a sparsely disseminated immunological component of the TME that have crucial roles in connecting innate and adaptive immune responses. DCs are the most effective antigen-presenting cells in living organisms, and can induce strong immune responses to tumor antigens [72]. Therefore, DCs are vital components in the immunostimulatory impact of RT. CAFs can hinder cytokine-induced differentiation of monocytes into DCs [73]. Further, CAFs induce a tolerogenic phenotype in mature DCs, evidenced by their diminished expression of activation markers (CD80, CD86, CD40, and HLA-DR) and reduced functional properties (migration, antigen uptake, and CD4 T cell priming). Interestingly, some of these effects disappear in conditioned medium from CAFs irradiated with fractionated medium-dose regimens (3 × 6 Gy); however, thymic stromal lymphopoietin and tryptophan 2,3-dioxygenase levels remain unaltered, suggesting that loss of CAF-mediated effects on DCs after ionizing radiation treatment does not rely on regulation of previously highlighted soluble mediators. Moreover, the changes described above only appeared following fractionated medium-dose RT, again suggesting an effect of the RT regimen applied on experimental results.

### T cells

I Specific CAF subsets show immunosuppressive abilities by influencing the recruitment, activation, migration, function, and differentiation of various T cell subpopulations in diverse cancers [74]. It was also confirmed that RT can dose-dependently modulate both the immune system and the TME. After a single dose, the quantity of tumor control and tumor reactive T cells increased with growing radiation dose [15]. In a study of liver metastases [75], recruitment of effector T cells to the tumor was increased after low-dose RT (LDRT), likely due to the noticeable decrease in CAFs (by >50%) after LDRT. Instead, A study by Gorchs et al. on NSCLC [76] found that CAF-conditioned medium exerted a strong immunosuppressive effect on activated T-cells, and that the effect remained after a single 18 Gy radiation dose. The secretion of relevant immunosuppressive molecules, such as prostaglandin E2, IL-6, IL-10, and TGF-β, was unchanged after irradiation; hence, the immunomodulatory effects of radiation on CAFs may vary depending on radiation dose.

### Natural killer cells

As the primary cytotoxic cells of the innate immune system, natural killer (NK) cells are the first line of resistance against tumor cells, and can perceive and eradicate senescent necrotic, infected, and malignant cells, which is important in antitumor responses. Following radiation, CAFs exert an immunosuppressive influence on NK cell cytotoxicity. In the co-culture experiments [77], melanoma-derived fibroblasts strongly inhibited NK cell cytotoxicity and cytokine production. CAFs can hinder NK cell activation by decreasing their proliferation rate, cytotoxic capacity, degree of degranulation, and expression of stimulatory receptors on the cell surface, while correspondingly improving the surface expression of inhibitory receptors [78]. Although single high-dose or fractioned RT regimens trigger enhanced surface expression of several checkpoint ligands on irradiated CAFs, such as significant upregulation of surface human leukocyte antigen E (HLA-E), PVR/CD155, and the activation receptor DNAM-1, they do not Gorses reverse CAF-mediated immunosuppressive effects against NK cells in vitro.

### Myeloid-derived suppressor cells

Myeloid-derived suppressor cells (MDSCs) are heterogeneous cell populations that strongly inhibit the anti-tumor activity of T and NK cells and stimulate regulatory T cells, leading to tumor progression. Co-culture of CAFs with tumor cells altered CCL7, CXCL1, CXCL2, and CXCL8 expression levels, and these chemokines promoted MDSC recruitment [79]. CCL2 secreted by CAFs improved MDSC recruitment in a murine liver cancer model [70] by binding CCR2 expressed on the surface of MDSCs, and was not observed in CCR2-deficient mouse models.

### Macrophages

Macrophages are mainly divided into two different types: “classical” activated (M1 or type I) and “alternative” activated (M2 or type II) [80]. M1 macrophages produce substantial amounts of pro-inflammatory cytokines and ROS, and coordinate T cell antitumor immune responses. In contrast, M2 macrophages produce immunosuppressors that promote tissue repair and angiogenesis and contribute substantially to tumor progression. There have been preliminary studies assessing the impact of radiation on macrophage polarization. There is considerable evidence that low-dose (single dose, ≤ 1.0 Gy) irradiation mainly induces anti-inflammatory activation of macrophages [81], while high-dose irradiation is more likely to enhance the pro-inflammatory properties of macrophages [82]. Radiation-induced CAF differentiation has recently been implicated in augmenting macrophage immunosuppressive function. In an NSCLC model [83], CAFs promoted changes in M0-macrophages that are compatible with both M1 and M2-phenotypes. Further, CAFs reduce the pro-inflammatory properties of M1-macrophages by inhibiting nitric oxide production, pro-inflammatory cytokines, migration, and M1-surface marker expression. Furthermore, although co-culture of irradiated or intact CAFs induces M0 macrophages to produce more CD80, CD163, CD206, IL-6, IL-10, and nitric oxide, neither single high dose nor fractioned regimens alter the immunomodulatory effects of CAFs on macrophages in vitro.

## Challenges and future directions

Given the complex and close relationships among CAFs, tumor cells, and immune cells, the development of effective treatment options will be a considerable challenge. While CAFs are understood to be fundamental in shaping responses to RT, some aspects of their effects have been incompletely elucidated.

First, various RT dose and fractionation regimens may have different effects on CAF function, as the reported effects of RT on CAFs have varied among studies, possibly due to differences in the radiation regimens (doses and fractions) used. Given the dual effects of RT on immunomodulation, specific RT regimens and combination therapies including RT should be carefully considered, to avoid undesirable immunosuppressive effects.

Second, differences in experimental models may have also influenced results. Steer et al. [84] investigated the impact of various CAFs on several tumor cell lines, under either direct or indirect co-culture conditions. The results revealed complex bi-directional direct and indirect interactions between cancer cells and CAFs, that influenced tumor progression and treatment efficacy. They observed that the effect of CAFs on tumor cell radiation response was largely reliant on the cell types and culture conditions used. This may be due to differences in the potential of tumor cells to activate fibroblasts to a CAF phenotype, leading to variation in fibroblast regulation of tumor progression and treatment resistance, which may generate difficulties in interpreting experimental data, particularly in preclinical investigations.

Third, for CAF focused approaches to be a feasible option, specific markers that can distinguish CAFs and evaluate their heterogeneity should be evaluated. CAFs are a functionally heterogeneous population in the TME, which may play contrasting roles. To date, the function of CAFs has been largely considered pro-tumoral. With the development of single-cell sequencing technology, information on CAF phenotypes and functional heterogeneity has increased, and the immune function of CAF may not always be pro-tumoral. Studies have shown that antigen-presenting cancer-associated fibroblasts in lung cancer can activate CD4 T cells to play the role of immune stimulation [85]. Similarly, tumor-suppressing subsets of CD105- CAFs have been found in pancreatic cancer, which effectively limit tumor growth in a way that dependent on functionally adaptive immunity and type 1 conventional DCs [86]. Anticancer subpopulations (cancer-restraining CAFs; rCAFs) are gradually recognized, however, their specific origins, molecular characteristics are still incomplete, and the mechanism of their immune effects are not clear, and comprehensive characterization of rCAFs warrants future study.

Furthermore, the significant effects of RT on CAFs and cross-talk between CAFs and the TME during RT remain largely unexplored. The number of studies on CAF-immune cell interactions is far from adequate, and the vast majority of the studies mentioned in our review did not determine the detailed cellular mechanisms by which CAFs influence immune cells. Therefore, an exhaustive investigation of the complex interaction between CAFs and the TME is warranted in the future, to facilitate recognition of CAF-mediated immune mechanisms following RT, and could inform development of approaches to stimulate unique antitumor reactions, in contrast to methods that simply inhibit and/or eradicate cancer cells.

## Conclusions

Taken together, research published to date indicates that CAFs affect multiple stages of cancer development, progression, metastasis, and treatment responses, and act to weaken the therapeutic effect of RT. The quantity of preclinical experiments aimed at restoring antitumor immune responses through CAF-targeted therapy has expanded substantially in recent years. Different therapeutic approaches are under investigation, including targeting of specific CAF functional subtypes, reprogramming CAFs to a tumor suppressor phenotype, or even switching between different functional subtypes. Research into CAFs is at a pivotal stage; nevertheless, how to enhance the immunostimulatory effect of CAFs after RT without causing negative immunosuppression effects remains a challenging question. Future studies will unravel the complex interactive relationships among CAFs, cancer cells, and immune cells, which will provide a strong framework for development of more effective cancer treatment regimens.
